# Molecular and Structural Characterizations of Lipases from *Chlorella* by Functional Genomics

**DOI:** 10.3390/md19020070

**Published:** 2021-01-28

**Authors:** Hajer Ben Hlima, Mouna Dammak, Aida Karray, Maroua Drira, Philippe Michaud, Imen Fendri, Slim Abdelkafi

**Affiliations:** 1 Laboratoire de Génie Enzymatique et de Microbiologie, Equipe de Biotechnologie des Algues, Ecole Nationale d’Ingénieurs de Sfax, Université de Sfax, Sfax 3038, Tunisia; hajer.benhlima@enis.tn (H.B.H.); dammakmouna23@yahoo.fr (M.D.); 2 Laboratoire de Biochimie et de Génie Enzymatique des Lipases, Ecole Nationale d’Ingénieurs de Sfax, Université de Sfax, Sfax 3038, Tunisia; aida.karray@enis.tn; 3 Laboratoire de Biotechnologie Végétale Appliquée à l’Amélioration des Cultures, Faculté des Sciences de Sfax, Université de Sfax, Sfax 3038, Tunisia; driramaroua@yahoo.fr (M.D.); imen.fendri@fss.usf.tn (I.F.); 4 CNRS, SIGMA Clermont, Institut Pascal, Université Clermont-Auvergne, F-63000 Clermont-Ferrand, France

**Keywords:** *Chlorella*, enzymes, lipases, molecular modeling

## Abstract

Microalgae have been poorly investigated for new-lipolytic enzymes of biotechnological interest. In silico study combining analysis of sequences homologies and bioinformatic tools allowed the identification and preliminary characterization of 14 putative lipases expressed by *Chlorella vulagaris*. These proteins have different molecular weights, subcellular localizations, low instability index range and at least 40% of sequence identity with other microalgal lipases. Sequence comparison indicated that the catalytic triad corresponded to residues Ser, Asp and His, with the nucleophilic residue Ser positioned within the consensus GXSXG pentapeptide. 3D models were generated using different approaches and templates and demonstrated that these putative enzymes share a similar core with common α/β hydrolases fold belonging to family 3 lipases and class GX. Six lipases were predicted to have a transmembrane domain and a lysosomal acid lipase was identified. A similar mammalian enzyme plays an important role in breaking down cholesteryl esters and triglycerides and its deficiency causes serious digestive problems in human. More structural insight would provide important information on the enzyme characteristics.

## 1. Introduction

The industrial enzymes market is estimated to be valued at USD 5.9 billion in 2020 and is projected to reach USD 8.7 billion by 2026, recording a Compound Annual Growth Rate (CAGR) of 6.5%, in terms of value [1]. The majority of enzymes currently used in industrial processes (more than 75%) are hydrolases [2]. Lipases represent the third most commercialized enzymes, after carbohydrases and proteases [3], and their production has constantly increased, they now account for more than one-fifth of the global enzyme market. The global Lipase Market size is anticipated to develop at a notable CAGR of about 7% over the calculated period from the current value of USD 0.6 billion in 2020. Lipases form an integral part of the industries ranging from biodiesels, food, nutraceuticals and detergents with little utilization in bioremediation, agriculture, cosmetics and leather [4].

Although lipases are produced by a huge number of organisms (bacterial, plant and animal origin), microbial lipases have attracted far more interest from researchers and industries than lipases from other sources, due to both their specific features and ease of production on large scale [5,6,7]. Notwithstanding current achievements, there is still a quest for lipases with improved and/or novel catalytic features like stability in harsh environments. Marine organisms can be an adequate source for such lipases as marine enzymes have demonstrated their useful for both process improvement and for the development of new process or products. Relevant types of lipases from marine organisms were identified and their novel features were discussed. They display, for example, salt tolerance, calcium independence and thermostable activities; they can also be stable in alkaline environment and were suggested to have antibiofilm action and higher catalytic efficiencies at temperatures lower than those from terrestrial microbial and/or mammal lipases [8]. However, few microalgal lipases and genes encoding lipases have been investigated and compared to bacterial, fungal, animal and plant lipases. In 2010, Demir and Tukel isolated and characterized for the first time a lipase from the photosynthetic cyanobacterium *Arthrospira. platensis* [9]. The lipase was a monomeric protein of 45 kDa with an isoelectric point of 5.9. It was specific for the 3-position in the ester bond. Godet et al. [10] isolated a new gene from the microalgae *Isochrysis galbana* encoding a 49 kDa lipase that shares similarities with fungal known lipase sequences. *Chlorella vulgaris* is a microalga belonging to the order of the *Chlorococcales*, which has a green color. It contains a significant number of intracellular proteins, carbohydrates, lipids, vitamin C, β-carotenes and B vitamins (B1, B2, B6 and B12), which is why it is commonly used for the preparation of food supplements. It is considered as raw materials for chemical compounds that have been affected by its primary and secondary metabolism, such as lipids, whose main application is for the generation of biodiesel [11]. This microalga has one of the highest lipid accumulating abilities in microalgae (50% of its DW), very high volumetric lipid productivity (VLP) of about 80 mg/L.day with a high growth rate in large-scale outdoor cultivation systems. Genetic manipulation technique for this microalga has already been established, showing great promise for improving its oleaginous phenotype by metabolic engineering [12]. Recently, its whole genome sequence was revealed by next-generation sequencing technologies, and the major metabolic pathways were identified [13]. Lipid metabolism has also been analyzed in multi-omics studies, including transcriptomics and proteomics to obtain the mechanistic insight of its lipid biosynthesis [14]. However, the TAG lipases have not been investigated yet. It will be of great importance to estimate the number and characteristics of its lipases, attracting knockdown targets for enhancement of lipid productivity. Here, a bioinformatic screening of a *C. vulgaris* genome was done to explore the presence of genes encoding putative lipases. The potential properties of the candidates are discussed on the basis of their three-dimensional (3D) model structures.

## 2. Results

### 2.1. Sequence Retrieval

The results of the amino acid sequence search showed that 14 protein sequences from nine *C. vulgaris* strains of the UTEX259 UTEX259 culture collection (taxid 3077)-scaffolds met determined criteria. The accession numbers of Transcriptome Shotgun Assembly (TSA) and Whole Genome Shotgun (WGS) sequences are given in Table 1andTable 2, respectively. As can be seen from the Table 1, all found lipase sequences belong to AB_hydrolase family (Interpro number IPR029058) and display Acyl hydrolase motif GXSXG. Nine of them show high sequence identity to Lipase_3 domain-containing protein (*Chlorella variabilis*) from the ESTHER database. Two sequences, namely Lip_5800 and Lip_5999, present high identity with sn1-specific diacylglycerol lipases alpha from *Auxenochlorella protothecoides* and *Micractinium conductrix*, respectively. In addition, 46.6% of sequence identity with chloroplastic Phospholipase A1 from *M. conductrix* was also detected with Lip_3448. Sequence homology analysis with multiple alignments revealed that these 14 sequences could be broadly clustered into two groups; 3 probable sn1-diacylglycerol lipases and 11 other lipase_3 family. Subsequently, gene prediction experiments were carried out with ab initio gene models (Table 2). These predictions showed different scaffold localization of the predicted lipase sequences with an exon number varying from 8 (Lip_5800 and Lip_5462) to 23 (Lip_2999). Lip_4551 and Lip_6297 lipases genes were found to be tandemly arrayed in the genome structure. These two genes have different sequence and size and their adjacent organization could allow faster transcription [15].

### 2.2. Physicochemical Characterization of Protein Sequences

ProtParam parameters shown in Table 3 reveal protein lengths varying from 421 to 1145 amino acids corresponding to diverse molecular masses (from 44.8 to 124.3 kDa). Various theoretical isoelectric points (Ip) were also found (4.09 to 9.34) and all proteins were predicted to have high molar extinction coefficients (46,300 to 193,210). Predicted repeats, motifs and localizations are given in Table 4. Among all predicted lipases, seven proteins have transmembrane motifs, including four predicted as being localized in plasma membrane and three in chloroplastic membrane. The seven other lipases have different cellular localizations (cytoplasmic, mitochondrian, chloroplastic or extracellular space), with five of them possessing a predicted signal peptide sequence. This enhances the possibility of extracellularity prediction however the signal peptides of chloroplasts and mitochondria are also N-terminal cleavable peptides [16]. They are less characterized than the secretory ones, but they are both rare in negatively charged amino acids and able to fold into amphiphilic α-helices [17].

The half-life is a prediction of the time it takes for half of the amount of protein in a cell to disappear after its synthesis in the cell; for all predicted lipases, it was found to be 30 h in mammalian (in vitro), more than 20 h in yeast, (in vivo) and more than 10 h in *Escherichia coli* (in vivo). ProtParam classifies also all studies proteins as stable (Instability index < 40).

Soluble predicted lipases have molecular weights between 44.8 and 102.5 kDa and Ip between 4.09 and 8.5. Concordant results were found by Ursu et al. [18]. The authors demonstrated, using the 2-DE profile of *C. vulgaris* soluble proteins, the presence of two protein groups that have been identified considering their isoelectrical points: a main group, having an Ip range of 4.0–5.5, and a minor group, with an Ip range of 6.0–8.0. However, the majority of separated proteins have apparent molecular weights range between 12 and 75 kDa. The difference observed herein could be explained by the fact that some proteins are not expressed under the culture conditions used by the authors.

### 2.3. D-Structural Modeling

The programs for 3D structural modeling automatically selected template structures mostly from fungal lipases as shown in Table S1 (PDBs: 6A0w, 6qpr, 4jei, 3o0d, 6unv, 4tgl, 3tgl). All models presented the typical α/β-hydrolase fold, with mostly parallel β-sheets, flanked on both sides by α-helixes. The highly conserved catalytic triad (serine, aspartic/glutamic acid and histidine) and the oxyanionic hole were well orientated in the space. The α/β hydrolase fold is one of the most thriving architectures in proteins across kingdoms, providing the skeleton for diverse enzymes [19] as well as an emerging class of non-catalytic but functionally important receptors [20]. Some of the predicted structures were very similar with the typical lipase motifs and are formed by one domain, but some other possesses an extra-transmembrane domain which could be quite bulky (Lip_4551 displays 9 helices against 4 for Lip_3928). Few membrane-bound lipases over intracellular or extracellular counterparts were studied. Recently the catalytic behavior of a membrane-associated lipolytic enzyme (MBL-Enzyme) from the microalgae *Nannochloropsis oceanica* was investigated by Savvidou et al. [21].

## 3. Discussion

TAG lipases responsible for the degradation of the lipids accumulated in oil bodies are attractive knockdown targets for the enhancement of the lipid productivity and storage in microalgae. Nonetheless, considering the numerous data available on bacterial, terrestrial plant and animal lipases those from algae and more especially microalgae have been relatively neglected. Therefore, more emphasize has to be given to the characterization of algal lipases, and hence, further work is needed in these aspects. Future approaches to maximize the enzymatic potential of microalgae are likely to focus on three different strategies: (i) the use of ever-increasing amounts of available omics data to optimize microalgal strains for the production of valuable products, through the overexpression of one or more enzymes by the use of genome editing tools; (ii) the identification and subsequent characterization of metabolic pathways involving the production of specific enzymes, such as lipases which are still poorly characterized; (iii) the search for genes with direct biotechnological applications in microalgal genomes and transcriptomes datasets. The feasibility of employing any of the aforementioned approaches or a combination of them will be directly influenced by progress in growth and genetic manipulation of microalgae.

**Figure 1:**
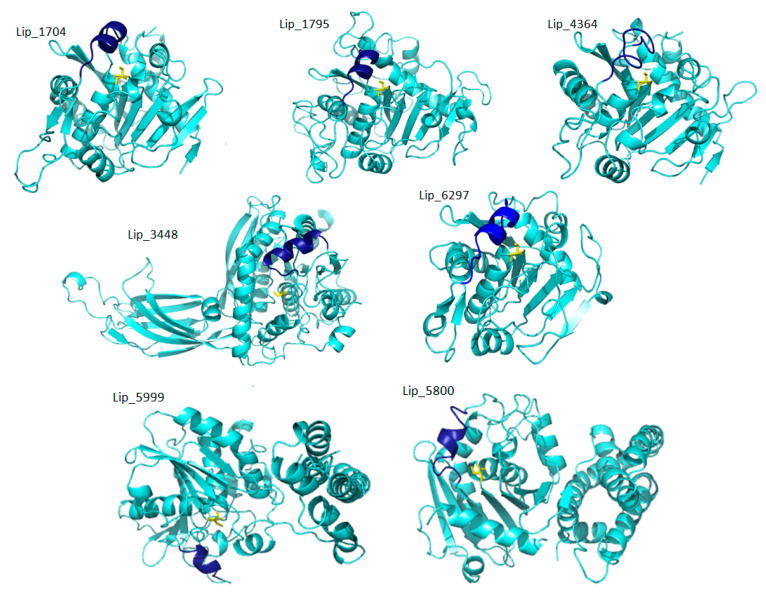
3D models of seven putative lipases without transmembrane domains. Four of them (Lip_1704, Lip_1795, Lip_4364, Lip_6297) display only the core module with a Rossman Fold architecture. Lip_3448 presents a C2 N-terminal domain, while Lip_5800 and Lip_5999 present a C-terminal alpha helices module. Lids are shown in dark blue and active site serine in yellow sticks.

**Figure 2:**
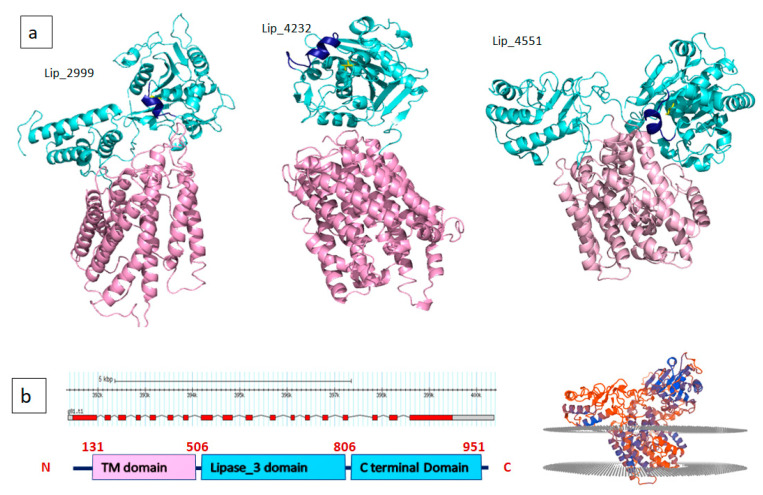
(**a**) Three predicted membrane-associated lipases with a transmembrane module shown in lilac; lids are shown in dark blue and active site serine in yellow sticks. (**b**) Gene annotation and domain boundaries of Lip_4551 (left panel), Qmeanbrane result for transmembrane localization for Lip_4551 (right panel).

In this study, we have used computational approach to identify lipase genes and to classify the respective lipases from a *C. vulgaris* strain. Lipases operate usually at the interface between lipid and water. An important feature of many lipases that is used for lipase classification is the presence of a mobile subdomain lid or flap located over the active site [22]. Among the 14 putative TAG lipases identified after *C. vulgaris* genome analysis, 10 have high identity in ESTHER database with Lipase_3 domain-containing protein. Family 3 of lipolytic enzymes are widely distributed in animals, plants and prokaryotes and possess the conserved consensus sequence GXSXG. Members of this family were demonstrated to be very closely related and exhibit the canonical α/β-hydrolase fold as well as the typical catalytic triad. Enzymes of this class exhibit also high activities at low temperature (less than 15 °C) believed to originate from a conserved sequence motifs they display [23]. Four lipases out of the 10 aforementioned were predicted to be either cytoplasmic, chloroplastic or extracellular. The six remaining could be anchored to a membrane with a distinct N-terminal transmembrane domain formed by at least four transmembrane helices (Figure 1 and Figure 2). Lip_4551 and Lip_2999 were predicted with quite similar 3D models composed of three domains: a catalytic domain containing the catalytic triad and a one helix lid, an N terminal transmembrane domain formed by long helices and a C terminal domain with mainly α helices (Figure 2). It has been reported that 10 additional modules can be attached to the core domain including lid modules, cap modules, N-terminal or C-terminal domains. Accordingly, superfamilies could be assigned to five groups (core, lid, cap, one additional domain or two additional domains) [24]. Predicted transmembrane domains by bioinformatic tools were already reported for microalga lipases [25]. Some authors characterized and used as a self-immobilized lipase for esterification reactions membrane bound lipase from microalga [21]. The membrane localization could be in intracellular or extracellular counterparts or even in lipid droplets (LD). In eukaryotes, some TAG lipases and their cofactors have been demonstrated to localize to LDs [26]. For example, Diatom Oleosome-Associated Protein 1 (DOAP1) is translocated from the ER to LDs in *Fistulifera solaris* [27].

As for Lip_3448, the N terminal module is a PLAT domain found in a variety of lipid-associated proteins. It forms a β-sandwich composed of two β-sheets of four β-strands each, which is known as a C2 domain in pfam classification. Interestingly, two predicted lipases have a C terminal module only composed of α helices. These two proteins (Lip_5800 and Lip_5999) are shown to be closely related in cladogram of sequence similarity. Hence, the predicted lipases could be classified into a main core with Rossman fold architecture lipases (Lip_1704, Lip_1795, Lip_4364, Lip_6297, Lip_5462, Lip_3928, Lip_4575), two domain lipases (Lip_4232, Lip_3448, Lip_3076, Lip_5800, Lip_5999) and three domain lipases including a transmembrane domain (Lip_2999 and Lip_4551).

**Figure 3:**
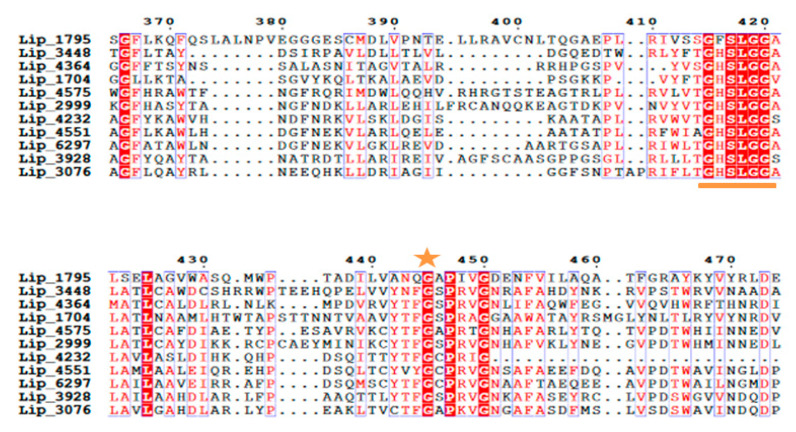
Multiple sequence alignment of putative lipases showing the conserved lipase 3 motif GXSXG and the conserved G residue for GX classification highlighted with orange star.

Oxyanion holes are crucial for high-energy oxyanion intermediate stabilization. They consist of two residues, which donate their backbone amide protons to stabilize the substrate in the transition state. In fact, during hydrolysis, a negatively charged tetrahedral intermediate is generated and the oxygen ion formed modulates the catalytic efficiency of the enzyme [28]. The first residue is located in the structurally conserved nucleophilic elbow. As a consequence, its backbone amide is positioned identically in all lipases. In contrast, the second oxyanion hole residue is not located in a region with conserved sequence and structure between lipases, but in a loop between the β3-strand and the αA-helix in the core module [29,30]. Consequently, lipases are classified into three classes according to their oxyanion hole type: GX, GGGX and Y [31]. In all lipases, the first oxyanion hole is a conserved glycine which contacts the nucleophilic elbow (highlighted with a star in Figure 3). When the oxyanion hole is formed by the amide backbone of the C-terminal neighbor X of this conserved glycine, it is termed as ‘GX type,’ with X being the second oxyanion hole residue. In our case, the inspection of the multiple sequence alignment of the 14 lipases demonstrates they belong all to the GX class with the conserved glycine (G) residue followed by an alanine (A), cysteine (C) or serine (S) residue (Figure 3). The lipases with GX oxyanion hole type are widely distributed and diverse, and they usually prefer hydrolyzing medium and long chain substrates [32]. The type of amino acid X is conserved inside the superfamilies; for example, it is hydrophilic in *Candida antarctica* like lipases (T), filamentous fungi lipases and cutinases (S, T), and hydrophobic in *Moraxella* (F), *Mycoplasma* (F, W) and *Pseudomonas* lipases (L, F, M) [29].

**Figure 4:**
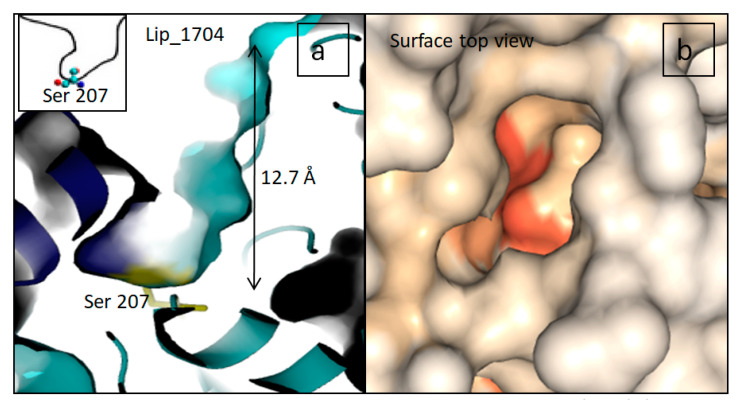
(**a**) Slabbed close up view of the active site cavity for Lip_1704 showing a crevice-like shape (**b**) A surface top view with DEPTH showing the shape of substrate entrance in the same protein.

According to the shape of the binding site cavity, lipases can be divided into three categories: (i) lipases with a funnel-like binding site (lipases from the mammalian pancreas and cutinase), (ii) lipases with tunnel-like binding sites (lipases from *Candida rugosa*, and *Candida antarctica* A) [33] and (iii) lipases with a crevice-like binding site (lipases from *Rhizomucor* sp. and *Rhizopus* sp.) [34]. It should be noted that most of the template structures used for 3D modeling are lipases from *Rhizomucor miehei.* In addition, the inspection of predicted open lid models like Lip_1704 showed a crevice-like cavity shape as shown in Figure 4.

**Figure 5:**
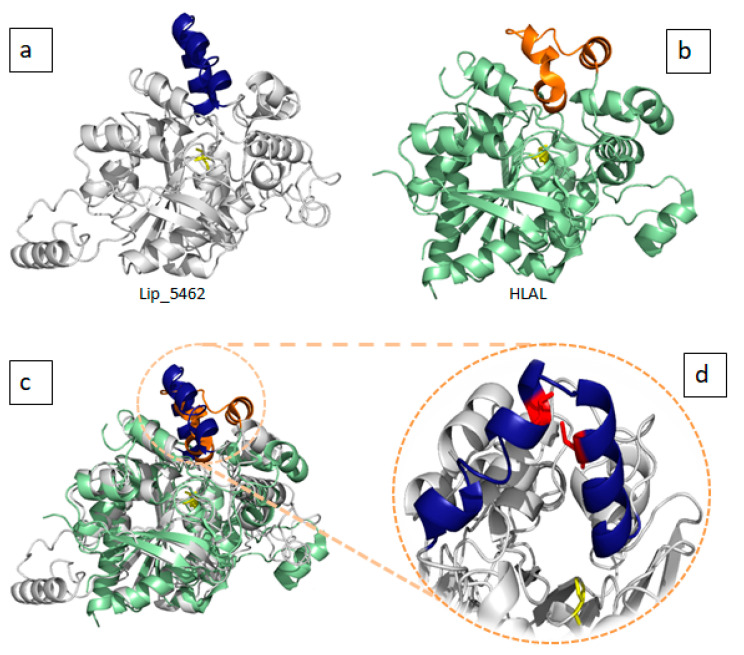
(**a**) 3D model of putative Lysosomal acid lipase Lip_5462 lid is shown in dark blue and catalytic serine in yellow sticks. (**b**) Crystal structure of human Lysosomal acid lipase PDB ID: 6V7N. Lid is shown in orange and catalytic serine in yellow sticks. (**c**) The overlay of the two aforementioned structures showing high structure similarities and lid differences. (**d**) A close up view of the three helices lid of Lip_5462 showing conserved cysteine residues in red sticks.

The amphipathic nature of the lid is crucial for the substrate specificity. It provides new insight into the structural basis of lipase substrate specificity and a way to tune the substrate preference of lipases. Based on the type of lid domain, lipases were also classified into three groups, such as lipases without lids, lipases with one loop or one helix lids and lipases with two or more helix lids. It has been reported that high temperature lipases contain larger lid domains with two or more helices, and that all mono- and diacylglycerol lipases have a small lid with a form of loop or helix [22]. As shown in Figure 1 and Figure 2, almost all lipases found in *C. vulgaris* have small lids with one loop (Lip_4364) or one helix (Lip_1704). However, Lip_5462 displays an entire cap domain with three small helices lid covering a deep cavity of 15.6 Å and shows, surprisingly, 40% of sequence identity with human lysosomal acid lipase (LAL). In fact, it has been demonstrated that, in addition to the direct association of lipases to oil bodies, macro-autophagy (referred to as lipophagy) plays a critical roles in lipid catabolism in eukaryotes [35]. During this type of autophagy, autophagosomes containing a portion of an oil body are merged with lysosomes containing LAL, which could contribute to TAG degradation [36]. Transcriptomic analysis of *Neochloris oleoabundans* (an oleaginous microalga) reveals up regulation of an LAL encoding gene under nitrogen starvation condition [37]. Accordingly, the in silico prediction method used for lipases of *C. vulgaris* allowed the identification of Lip_5264, which could be transported to lysosomes. This enzyme was predicted to have a signal peptide and 40% of sequence identity with the human LAL. It consists of a core domain belonging to the classical α/β hydrolase-fold family with a classical catalytic triad (Ser-161, His-378, Asp-347), an oxyanion hole and a “cap” domain, which probably regulates substrate entry to the catalytic site (Figure 5). LAL breaks down cholesteryl esters (CEs) and TGs into free cholesterol, glycerol and fatty acids (1–3). Defective LAL have been associated with two autosomal recessive diseases in humans: Wolman’s disease and CE storage disease [38,39]. The gene of Lip_5264 consists of 8 exons spread over almost 4 kb, while human LAL consists of 10 exons spread over 36 kb. Lip_5264 encodes a 445 amino acid mature protein following the cleavage of 24 signaling peptide residues, with an expected molecular mass of 50 kDa whereas human LAL encodes for 378 residues with a signal peptide of 21 amino acids and a molecular mass of 43 kDa. The two compared proteins are glycosylated and share high structure identity, as shown in Figure 5c with some differences, including the lid helices, which contain a cluster of highly conserved Cys residues C 236 and C 243 (Lip_5264 numbering) (Figure 5d). The lysosomal proteins in microalga have not yet been fully investigated, and it remains unclear how lipophagy contributes to lipid degradation. These should be an attractive research topic in a future work. Microalgae are a good source of nutrients for human nutrition. However, they are also rich in various biomolecules, which may have a potential in promoting human health. Defective or diminished LAL activity of human LAL has been associated with some mutations and the molecular mechanisms of these loss-of-function mutants leading to WD and CESD have yet to be explored. Some study demonstrated that these mutations could be located in the signal peptide or in the lid domain [40]. A complete physicochemical characterization of this *C. vulgaris* LAL combined with a deep structure–function relationship investigation of the probable mutation effect using a structure-based molecular model speculating the loss of function could be of interest. The current treatment options for CESD phenotypes are limited to diets excluding cholesterol and lipid-rich food, cholesterol lowering drugs such as statins and ultimately liver transplantation. Recombinant LAL replacement therapy has been shown to be effective in animal models and human clinical trials and was recently authorized in Europe and the United States [41].

## 4. Materials and Methods

### 4.1. Sequence Retrieval

BlastP search was performed using amino acid sequences of functionally characterized lipases from terrestrial plants (*Trifolium pretense* and *Diplocarpon rosae*), fungi (*Colletotrichum chlorophyti*), microalga (*Scenedesmus* sp. and *Symbiodinium microadriaticum*) and bacteria (*Pseudomonas fluorescens* and *B. subtilis*) available in the NCBI database (http://ncbi.nlm.gov/protein/). The FASTA sequences were searched using tblastn modality against Transcriptome Shotgun Assembly database (TSA) of *C. vulgaris* strain UTEX259 UTEX259 (taxid 3077) and every hit with an E-value < 10^−5^ was identified as putative Lipase transcript. The open reading frames (ORFs) were searched using the ORF finder program [42] and the longer ones were blasted a second time against non-redundant protein database to ensure that the respective TSA corresponds to a putative Lipase ORF. The selected TSA sequences were submitted to a blastn search against the whole Genome Shotgun contigs (WGS) database of the same *C. vulgaris* strain (taxid 3077) and single hits with E-value < 10^−100^ were identified as scaffolds with putative Lipase genes. Gene predictions from the selected WGS scaffolds were performed using ab initio gene models through Augustus [43]. The application was trained on the gene structures of *Chlamydomonas reinhardtii* and the TSA sequences were used in cDNA uploaded option. The final output ORF and protein sequences were saved for further in silico analysis.

### 4.2. Multiple Sequence Alignment

The multiple sequence alignment and calculation of cladogram illustrating sequence similarity relationships among the 14 putative lipase sequences was executed by MAFFT (v7.310) with G-INS-1 strategy, unalign level 0.8, leave gappy region options for alignment and UPGMA as average linkage method for clustering. Rendering was done using ESPript [44].

### 4.3. Physicochemical Characterization of Protein Sequences

Basic physicochemical properties such as molecular weight, extinction coefficient, isoelectric point, aliphatic index, grand average of hydropathicity and instability index were estimated by ProtParam tool (http://web.expasy.org/protparam/) [35]. Extinction coefficients were calculated assuming all pairs of Cys residues form cystines or assuming all Cys residues are reduced. Sequence analysis and lipase motifs search were performed with InterPro [45] and the Expasy my hits search tool (https://myhits.isb-sib.ch/cgi-bin/motif_scan), respectively. These sequences were also compared in the ESTHER database to check higher sequence identity [19]. For predicting subcellular localization Deepmito [46], Mitoprot v1.101 [47], HECTAR v1.3 [48] and TMHMM v2.0 [49] were performed. Putative signal peptides in each sequence were predicted using the SignalP 4.0 server [50]. Since N-glycosylation was widly described for lipases prediction of N-glycosylation sites were performed using NetOGlyc 4.0 Server [51].

### 4.4. Tertiary Structure Prediction, Structure Validation and Quality Prediction

Three-dimensional models of the selected putative enzymes were generated using different approaches. For sequences with acceptable homology in the template of the programs, UCSF Chimera (https://www.rbvi.ucsf.edu/chimera/) and the automated protein homology modeling server SWISS-MODEL (http://swissmodel.expasy.org/) were used. For sequences with low homology with the structures in the database, multiple-threading alignments using the I-TASSER approach (zhanglab.ccmb.med.umich.edu/I-TASSER/) was used. I-TASSER is an automated bioinformatics tool for predicting protein structures from an amino acid sequence followed by iterative structural assembly simulations and atomic-level structure refinement.

The predicted structures were evaluated to ensure correctness of the model stereochemistry, as checked by a Ramachandran plot (http://mordred.bioc.cam.ac.uk/~rapper/rampage.php) (Lovell et al., 2003) and Verify 3D [52]. The Ramachandran plot scores of the predicted structures showed more than 90% of the amino acids were in favorable regions. ProSA-web Z-score plot (https://prosa.services.came.sbg.ac.at/prosa.php) [53] was used to check whether the Z-score of the input structures is within the range of typically found for the native proteins of a similar size. The Z-score values of all protein structures checked in this study were highlighted as a black dot, which indicates being in the range of native conformations. The final modeled structures were further energetically minimized and molecular dynamics simulation was performed with CABS-flex 2.0 (http://212.87.3.12/CABSflex2). The latter program is an efficient simulation engine that allows modeling of the large-scale conformational change related to protein flexibility [54]. The models were comprehensively analyzed using PyMol (http://pymol.org/) to check for the presence of a lid, and the existence and orientation of the catalytic triad. The depth of the putative intramolecular tunnels was calculated with DEPTH [55] taking residues from the oxyanion hole in each candidate as the cavity end point.

## 5. Conclusions

Genomic mining by combining bioinformatics analysis and functional screening provides opportunities to find out novel biocatalysts, such as lipases. The present study allowed the in silico characterization of 14 putative *C. vulgaris* lipases with different cellular localization. Membrane associated lipases were also detected and described for the first time in this species. The 14 lipases display an acyl hydrolase motif (GXSXG) and belong to the α/β hydrolase lipase 3 family and GX class. These putative lipases could be candidates for metabolic engineering in a future study to improve this microalga lipid productivity. In this study, we also report, for the first time, a putative lysosomal acid lipase produced by a green microalga. Further investigation on the generated 3D models, such as docking studies and MD simulations, will provide important information on the substrate catalytic process and the binding characteristics and could be of interest to understand molecular mechanisms of the loss-of-function mutants leading to WD and CESD in humans.

## Figures and Tables

**Table 1:** Putative TAG lipases in *C. vulgaris* with their sequence-based motif and family search.

TSA ID	Family InterPro	Family Pfam	Acyl Hydrolase Motif (GXSXG)	Highest Identity in ESTHER Database	Accession Number ESTHER Database
GHLX01005462.1	IPR029058, AB_hydrolase	PF04083, Abhydro_lipase	GHSQG	62.2% Lipase (*M. conductrix*)	A0A2P6V8F3
GHLX01003448.1	IPR029058, AB_hydrolase IPR002921, Fungal_lipase-like	PF01764, Lipase_3	GHSLG	46.6% Phospholipase A(1) chloroplastic (*M. conductrix*)	A0A2P6VDJ3
GHLX01004364.1	IPR029058, AB_hydrolase IPR002921, Fungal_lipase-like	PF01764, Lipase_3	GHSLG	79.9% Lipase_3 domain-containing protein (*C. variabilis*)	E1ZB31
GHLX01003076.1	IPR029058, AB_hydrolase IPR002921, Fungal_lipase-like	PF01764, Lipase_3	GHSLG	41.5% Lipase_3 domain-containing protein (*C. variabilis*)	E1ZMR0
GHLX01002999.1	IPR029058, AB_hydrolase IPR002921, Fungal_lipase-like	PF01764, Lipase_3	GHSLG	56.8% Lipase_3 domain-containing protein (*C. variabilis*)	E1Z559
GHLX01001704.1	IPR029058, AB_hydrolase IPR002921, Fungal_lipase-like	PF01764, Lipase_3	GHSLG	56.1%Lipase_3 domain-containing protein (*C. variabilis*)	E1ZAU0
GHLX01004551.1	IPR029058, AB_hydrolase IPR002921, Fungal_lipase-like	PF01764, Lipase_3	GHSLG	53.5% Lipase_3 domain-containing protein (*C. variabilis*)	E1Z6D5
GHLX01003928.1	IPR029058, AB_hydrolase IPR002921, Fungal_lipase-like	PF01764, Lipase_3	GHSLG	54.5% Lipase_3 domain-containing protein (*C. variabilis*)	E1ZMR0
GHLX01006297.1	IPR029058, AB_hydrolase IPR002921, Fungal_lipase-like	PF01764, Lipase_3	GHSLG	61.7% Lipase_3 domain-containing protein (*C. variabilis*)	E1Z6D6
GHLX01001795.1	IPR029058, AB_hydrolase IPR002921, Fungal_lipase-like	PF01764, Lipase_3	GFSLG	60.8% Lipase_3 domain-containing protein (*C.a variabilis*)	E1Z814
GHLX01004575.1	IPR029058, AB_hydrolase IPR002921, Fungal_lipase-like	PF01764, Lipase_3	GHSLG	55.3% Lipase_3 domain-containing protein (*C. variabilis*)	E1Z559
GHLX01004232.1	IPR029058, AB_hydrolase IPR002921, Fungal_lipase-like	PF01764, Lipase_3	GHSLG	52.4% Alpha beta-hydrolase (*C. sorokiniana)*	A0A2P6TJS1
GHLX01005999	IPR029058, AB_hydrolase IPR002921, Fungal_lipase-like	PF01764, Lipase_3	GHSLG	62.2% *sn*1-specific diacylglycerol lipase alpha (*Auxenochlorella protothecoides*)	A0A087SMB1
GHLX01005800	IPR029058, AB_hydrolaseIPR002921, Fungal_lipase-like	PF01764, Lipase_3	GHSLG	44.5% *sn*1-specific diacylglycerol lipase alpha (*M. conductrix*)	A0A2P6V840

**Table 2:** Genes annotation of the putative TAG predicted lipases.

Transcriptome Shotgun Assembly ID	Genome Survey Sequences ID	Start	End	Gene Length	Strand	5′ UTR	3′ UTR	StartCodon	StopCodon	Exon Number
GHLX01005462.1	VATW01000002.1	1,249,709	1,253,360	3651	+	1,249,709	1,253,181	1,249,877	1,253,178	8
GHLX01003448.1	VATW01000019.1	480,480	486,471	5991	+	480,893	485,948	480,896	485,947	15
GHLX01004364.1	VATW01000012.1	444,856	447,981	3125	−	447,981	444,884	447,869	444,885	9
GHLX01003076.1	VATW01000017.1	300,534	306,599	6065	−	306,599	300,680	306,266	300,681	16
GHLX01002999.1	VATW01000004.1	234,154	243,389	9235	−	243,389	235,621	243,213	235,622	23
GHLX01001704.1	VATW01000077.1	53,966	57,537	3571	+	54,324	57,537	54,485	57,503	12
GHLX01004551.1	VATW01000014.1	391,368	400,369	9001	+	391,465	400,369	391,466	399,488	18
GHLX01003928.1	VATW01000021.1	364,412	371,783	7371	+	364,615	371,704	364,616	371,701	17
GHLX01006297.1	VATW01000014.1	387,248	391,356	4108	+	387,638	391,356	387,830	391,200	10
GHLX01001795.1	VATW01000003.1	1,009,232	1,012,963	3731	+	1,009,437	1,012,963	1,009,559	1,012,785	9
GHLX01004575.1	VATW01000004.1	243,414	249,169	5755	−	248,807	243,564	248,803	243,565	19
GHLX01004232.1	VATW01000004.1	387,912	396,833	8921	+	388,099	393,753	388,100	393,622	16
GHLX01005999.1	VATW01000002.1	467,247	474,411	7164	−	474,331	467,332	474,153	467,333	16
GHLX01005800.1	VATW01000021.1	56,136	59,601	3465	+	56,233	59,492	56,234	59,489	8

**Table 3:** ProtParam parameters of the predicted lipases.

Protein Name	Length (Amino Acids)	Molecular Mass (Da)	Theoretical Ip	Total Number of Negatively Charged Residues (Asp + Glu)	Total Number of Positively Charged Residues (Arg + Lys)	Molar Extinction (M^−1^ cm^−1^)	Half-Life	Grand Average of Hydropathicity Index (GRAVY)
Lip_5462	469	50,526.70	6.94	35	34	58,830 58,330	30 h (mammalian reticulocytes, in vitro), >20 h (yeast, in vivo), >10 h (*Escherichia coli*, in vivo).	0.056
Lip_3448	810	87,834.98	4.50	125	55	113,955 113,330	30 h (mammalian reticulocytes, in vitro), >20 h (yeast, in vivo), >10 h (*Escherichia coli*, in vivo).	−0.223
Lip_4364	433	47,169.73	6.08	35	28	105,475 104,850	30 h (mammalian reticulocytes, in vitro), >20 h (yeast, in vivo), >10 h (*Escherichia coli*, in vivo).	0.083
Lip_3076	966	104,984.87	8.78	80	91	164,875 163,750	30 h (mammalian reticulocytes, in vitro), >20 h (yeast, in vivo), >10 h (*Escherichia coli*, in vivo).	0.086
Lip_2999	1104	121,199.69	8.67	98	110	193,210 191,710	30 h (mammalian reticulocytes, in vitro), >20 h (yeast, in vivo), >10 h (*Escherichia coli*, in vivo).	−0.125
Lip_1704	421	44,824.88	8.57	29	35	47,050 46,300	30 h (mammalian reticulocytes, in vitro), >20 h (yeast, in vivo), >10 h (*Escherichia coli*, in vivo).	0.057
Lip_4551	1145	124,302.95	7.43	103	103	157,425 156,300	30 h (mammalian reticulocytes, in vitro), >20 h (yeast, in vivo), >10 h (*Escherichia coli*, in vivo).	−0.057
Lip_3928	934	100,739.17	8.92	80	93	132,905 131,780	30 h (mammalian reticulocytes, in vitro), >20 h (yeast, in vivo), >10 h (*Escherichia coli*, in vivo).	−0.013
Lip_6297	530	56,818.32	6.12	51	47	69,940 69,440	30 h (mammalian reticulocytes, in vitro), >20 h (yeast, in vivo), >10 h (*Escherichia coli*, in vivo).	0.101
Lip_1795	557	59,817.51	4.09	67	23	97,330 96,830	30 h (mammalian reticulocytes, in vitro), >20 h (yeast, in vivo), >10 h (*Escherichia coli*, in vivo).	0.110
Lip_4575	726	81,163.18	9.26	67	28	162,885 162,260	30 h (mammalian reticulocytes, in vitro), >20 h (yeast, in vivo), >10 h (*Escherichia coli*, in vivo).	−0.117
Lip_4232	779	85,389.08	9.34	62	83	107,675 106,800	30 h (mammalian reticulocytes, in vitro), >20 h (yeast, in vivo), >10 h (*Escherichia coli*, in vivo).	0.082
Lip_5999	1003	102,522.28	5.31	112	86	85,425 84,800	30 h (mammalian reticulocytes, in vitro), >20 h (yeast, in vivo), >10 h (*Escherichia coli*, in vivo).	−0.143
Lip_5800	629	67,075.99	4.98	98	66	58,160 57,410	30 h (mammalian reticulocytes, in vitro), >20 h (yeast, in vivo), >10 h (*Escherichia coli*, in vivo).	−0.307

**Table 4:** Predicted repeats, motifs and localization of putative lipases.

Protein Name	Predicted Localization	Signal Peptide Sequence	Membrane Helix	N Glycosylation Sites
Lip_5462	Ps (extracellular space)	MNVGRVAALFACLLQGACLALAVQ	-	325
Lip_3448	Mito	-	-	170
Lip_4364	Ps (extracellular space)	MRPAITEALLAVLVCLVVGANGA	-	134/180/273/280
Lip_3076	Chloro (membrane)	-	42–64/84–106/129–151/161–183/266–288/314–336/348–370	8/676
Lip_2999	Chloro (membrane)	-	120–142/162–184/203–225/245–267/293–315/341–363/400–422	187/349/383/1030
Lip_1704	Chloro	MKLGLPLLLAALLLAAAAPATAR	-	230/260/305/369
Lip_4551	Chloro (membrane)	-	139–161/176–198/222–244/254–276/313–330/362–384/411–433/453–475/482–504	279/946
Lip_3928	plasma membrane	-	62–84/174–196/220–242/254–276	-
Lip_6297	Ps (extracellular space)	MFIRVQSRVVSAVFTAIIFSLLFMSLVPTLQGN		392
Lip_1795	cyto	-	-	19/53/307
Lip_4575	plasma membrane	MYIANTSVGGVLTLASFAMLAHGL	6–28/48–70/80–102/115–137/170–189/196–218	5
Lip_4232	plasma membrane	-	31–53/66–88/108–130/145–167/202–224/251–273/300–322	475
Lip_5999	chloro	-	-	-
Lip_5800	chloro	-	-	487

## Supplementary Materials

The following are available online at https://www.mdpi.com/1660-3397/19/2/70/s1, Table S1 Templates used for 3D model generation.
