# Supplementary material for: Molecular and Structural Characterizations of Lipases from Chlorella by Functional Genomics
Source: Mar Drugs. 2021 Jan 28;19(2):70. doi: 10.3390/md19020070 (PMC7910983; doi:10.3390/md19020070)
Supplement: Supplementary file 1 [file marinedrugs-19-00070-s001.pdf]

Table S1. T**emplates used for 3D model generation**

Protein name	Template PDB ID	Template protein	Prediction program	Identity	Domain number	Ramachandran Favoured
Lip_5462	6v7n.1.A	Human Lysosomal Acid Lipase	Swiss-model	39.61%	1	92.27%
Lip_3448	6A0w.A	lipase from Rhizopus microsporus var. chinensis	Swiss-model	37.7%	2	95.20%
Lip_4364	6A0w.A	lipase from Rhizopus microsporus var. chinensis	Chimera	31.89%	1	91.59%
Lip_3076	6qpr.1.A	Rhizomucor miehei lipase propeptide complex, Ser95/Ile96 deletion mutant	I-Tasser	26.18%	2	92.12%
Lip_2999	6qpr.1.A	Rhizomucor miehei lipase propeptide complex, Ser95/Ile96 deletion mutant	I-Tasser	25.6%	3	94.32%
Lip_1704	3tgl.1.A	RHIZOMUCOR MIEHEI TRIACYLGLYCERIDE LIPASE	Chimera	35%	1	94.57%
Lip_4551	3o0d.A	Crystal structure of Lip2 lipase from Yarrowia lipolytica at 1.7 A resolution	Chimera	33.5%	3	95.23%
Lip_3928	4tgl.1.A	Triglyceride lipase from Rhizomucor miehei	I-Tasser	26.36%	1	95.23%
Lip_6297	3ngm.1.A	lipase from Gibberella zeae	I-Tasser	21.4%	1	95.20%
Lip_1795	6qpr.1.A	Rhizomucor miehei lipase propeptide complex, Ser95/Ile96 deletion mutant	I-Tasser	21.6%	1	92.76%
Lip_4575	3ngm.1.A	lipase from Gibberella zeae	Chimera	29.24%	2	94.60%
Lip_4232	6A0w.A	lipase from Rhizopus microsporus var. chinensis	Swiss-model	38.8%	2	95.44%
Lip_5999	6unv.1.A	lipase/esterase from the fungus Rasamsonia emersonii	I-Tasser	21.89%	2	94.42%
Lip_5800	6qpr.1.A	Rhizomucor miehei lipase propeptide complex, Ser95/Ile96 deletion mutant	I-Tasser	20.7%	2	94.26%
